# Failure risk in post-earthquake evacuation and logistics in Padang City

**DOI:** 10.4102/jamba.v17i1.1773

**Published:** 2025-08-15

**Authors:** Bayu M. Adji, Bambang Istijono, Muhazir Rahendra, Suhana Koting, Abdul Hakam, Taufika Ophiyandri, Masrilayanti Masrilayanti, Rezko Yunanda

**Affiliations:** 1 Department of Civil Engineering, Faculty of Engineering, Universitas Andalas, Padang, Indonesia; 2 Department of Natural Resources Management, Universitas Andalas, Padang, Indonesia

**Keywords:** evacuation, logistics, earthquake, tsunami, disaster risk, Padang City, FMEA, communication system

## Abstract

This study examines the challenges and risks in managing evacuations and distribution logistics after tsunamis triggered by earthquakes, focusing on Padang City. Key issues include inadequate evacuation routes, delayed information dissemination and inefficient logistical coordination, compounded by the city’s topography. Data were collected from 20 key informants, structured interviews and questionnaires involving stakeholders with direct operational experience in disaster response, such as the Regional Disaster Management Agency, the Transportation Office and the Social Affairs Office. The failure mode and effect analysis (FMEA) method identified and prioritised potential failure modes based on risk priority number (RPN) values. The analysis revealed that the most critical risks in evacuation include damaged evacuation routes, lack of public knowledge about safe routes and heavy traffic congestion. The most significant dangers for logistics were post-earthquake telephone network disruptions, long delivery times and vehicle capacity limitations. These risks were found to hinder practical response efforts significantly. The study recommends targeted mitigation strategies such as strengthening communication systems, improving evacuation route infrastructure and increasing preparedness among communities and authorities. These findings offer practical insights to enhance disaster risk reduction and emergency logistics planning in coastal cities vulnerable to tsunamis.

**Contribution:**

Research addresses critical gaps in tsunami evacuation and logistics management by analysing risks using FMEA techniques. The study highlights gaps in infrastructure, social dynamics and policies on urban resilience and disaster risk reduction. By emphasising practical strategies to mitigate key risks such as communication breakdowns and logistics coordination, the study provides actionable insights to improve disaster preparedness and response.

## Introduction

**FIGURE 1:**
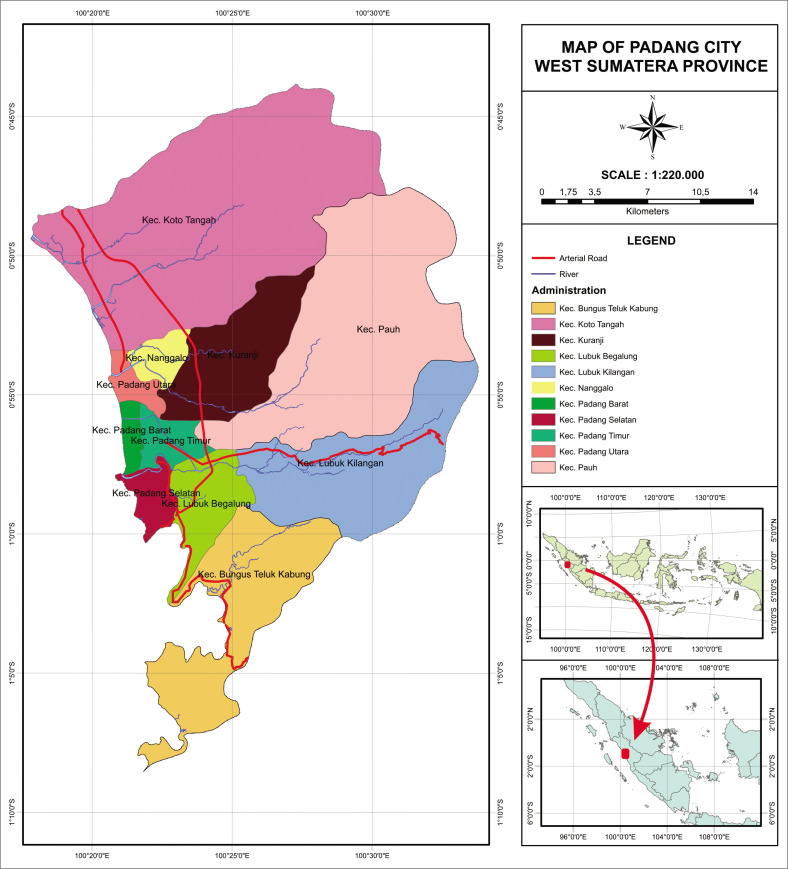
Map of Padang City.

Padang City, located on the west coast of Sumatra Island in West Sumatra province, covers an area of 695 km^2^. Approximately 20% of the area is designated as utilised land, while 80% serves as protected zones. The city’s elevations vary significantly, ranging from 25 m to 1500 m above sea level. Padang has an estimated population of 915 000 residents. The spatial layout of Padang City is depicted in Figure 1.

**FIGURE 2:**
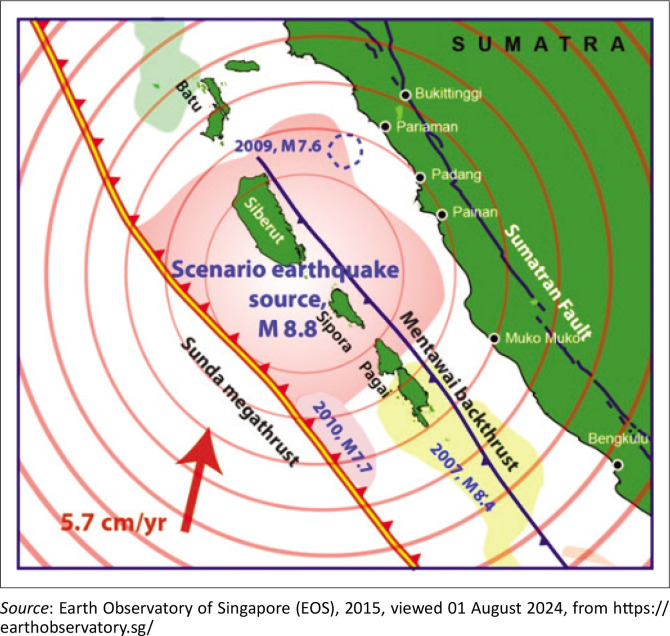
The section of the Sunda megathrust fault (8.8 SR scenario).

Padang is particularly vulnerable to natural hazards because of its position near the boundary of the Indo-Australian and Sunda (Eurasian) tectonic plates, which converge at approximately 5.70 cm per year. This tectonic interaction along the Sunda megathrust has made the region highly susceptible to large earthquakes and tsunamis. Historical records indicate that Padang and its surrounding areas have experienced several devastating earthquakes. For example, the subduction earthquakes of 1797 and 1833 triggered destructive tsunamis that caused widespread damage along the western coast of Sumatra. The 1833 tsunami reached far inland, including parts of Padang, and affected thousands of residents. More recently, the 2004 Indian Ocean earthquake, although its epicentre was located off the coast of Aceh, marked a turning point in how tsunami risks were perceived across Indonesia, which prompted a nationwide reassessment of coastal vulnerability. Padang experienced further seismic activity in the following years, including significant earthquakes in 2007, 2009 and 2010, reaffirming and validating the seismic vulnerability of this region (DKKV 2010). Scientific research predicts an 8.8 magnitude earthquake on the Sunda megathrust (Figure 2) that could trigger a giant tsunami, seriously threatening communities across West Sumatra, including Padang, Pariaman and Painan.

A disaster evacuation plan outlines designated routes to guide evacuees from areas at risk to locations of safety. It involves planning the evacuation routes and efficiently moving populations during emergencies. (Ismail, Masrilayanti & Anggraini 2020; Kim, Park & Kim 2023; Menghoung et al. 2023). In 2017, in coordination with disaster management stakeholders, the Padang city government developed a contingency plan in anticipation of a magnitude 8.8 earthquake, followed by a tsunami. The scenario assumes the earthquake would originate 150 km southwest of Padang at a depth of 30 km, occurring during Monday morning rush hour. According to the plan, the first tsunami wave, ranging between 8 m and 12 m in height, would hit Padang’s coastline within 20 min and extend 2 km – 3 km inland within 3–4 h. It is estimated that approximately 273 000 of the city’s 915 000 residents would be directly affected (Government of Padang 2017).

**FIGURE 3:**
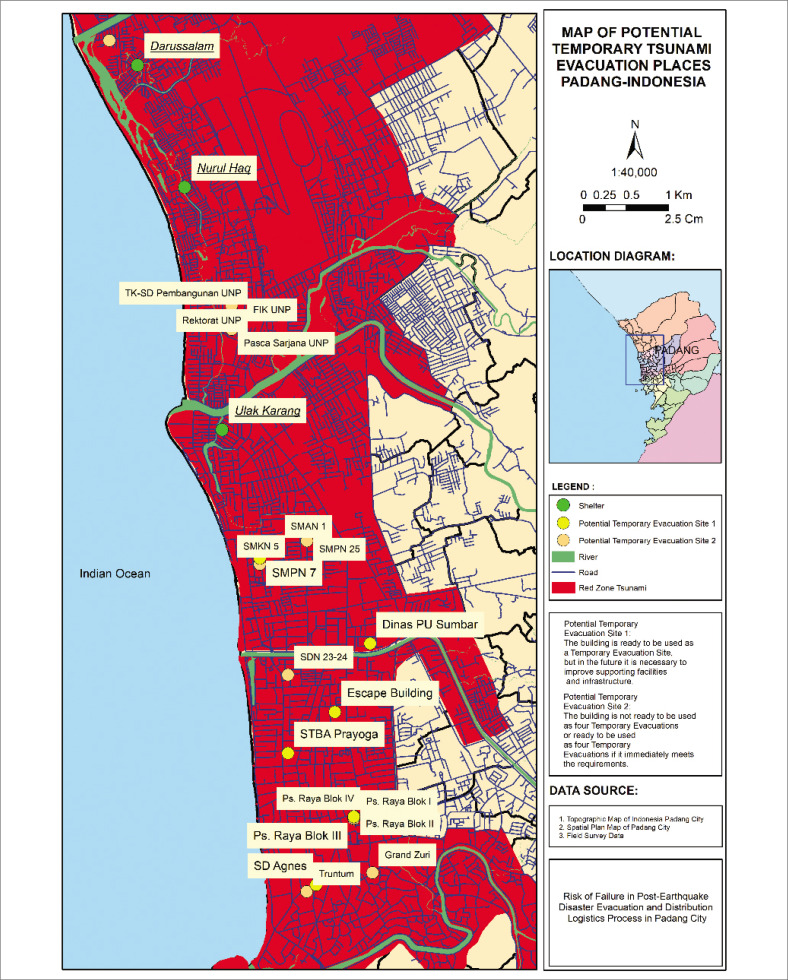
Map showing temporary evacuation shelters and tsunami-prone zones in Padang City.

Evacuation strategies for the Padang coast include horizontal evacuation (moving to tsunami-free zones) and vertical evacuation to nearby multi-storey shelters. Currently, the city has three designated temporary evacuation shelters: Darussalam (Bungo Pasang), Nurul Haq (Parupuk Tabing) and Wisma Indah (Ulak Karang) (Ophiyandri et al. 2022). Additional tall buildings could potentially expand the number of viable evacuation sites. However, spatial analysis shows that existing and potential evacuation buildings are heavily concentrated in central Padang. At the same time, the northern and southern areas (classified as tsunami red zones) lack suitable buildings for vertical evacuation (Figure 3).

**FIGURE 4:**
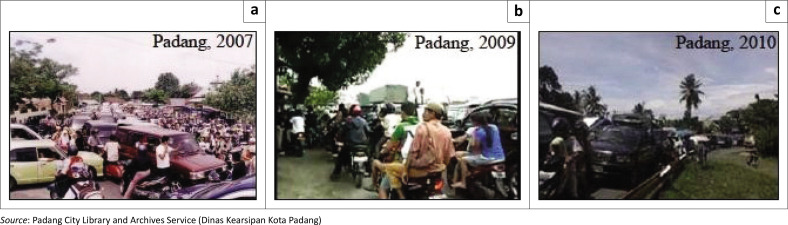
Traffic jams during the earthquake in the city of Padang. (a) Earthquake, 6 March 2007 in Padang City; (b) Earthquake, 30 September 2009 in Padang City; (c) Earthquake, 25 October 2010 in Padang City.

During the 2007, 2009 and 2010 earthquakes, traffic congestion severely hindered evacuation efforts as many residents attempted to flee using motorbikes and private vehicles. Numerous accidents occurred, and the existing evacuation routes proved insufficient to handle the volume of evacuees. Because vertical evacuation had not yet been integrated into the emergency response strategy, most residents fled horizontally towards higher ground. This approach resulted in severe congestion, longer evacuation times and increased confusion and panic among evacuees, as seen in Figure 4. Despite a limited public understanding of early warning systems, there remained a general trust in the government to provide accurate information following seismic events (Bae et al. 2021; Lozano & Taboada 2021; Munawar et al. 2021b).

Increased fatalities and damage were because of the absence of integrated evacuation routes, delays in evacuation responses and information dissemination and inefficient transportation system use. Disaster mitigation is essential to analyse potential failures in community evacuation processes and minimise disaster risks and impacts (Kang et al. 2021; Noviana et al. 2021).

In addition to evacuation challenges, post-earthquake conditions in Padang highlight severe logistical issues in delivering aid. Although evacuation and aid distribution occur at different disaster response stages, they are interconnected and mutually dependent. Failures in evacuation infrastructure can delay aid access, while ineffective logistical coordination can hinder recovery efforts for evacuees. Therefore, analysing the evacuation phase and post-disaster distribution logistics is essential for understanding risk management and enhancing overall disaster response effectiveness.

In post-disaster logistics, government-established institutions ensure the fair fulfillment of community rights as per Law No. 24 of 2007 on disaster management. The government and regional authorities must protect and aid communities during disasters, including providing timely logistical support. However, challenges or common issues such as damaged road infrastructures, collapsed bridges and delays in logistics emerged as effective aid distribution disruptors (Redi et al. 2021; Rofiah, Kawai & Hayati 2024). In addition, inadequate supervision of consumable aid storage leads to quality degradation because of prolonged storage periods. Limited goods availability, distribution timing and transportation facilities complicate aid delivery. These challenges create uncertainties and risks within disaster aid logistics operations (Ariyachandra & Wedawatta 2023; De Veluz et al. 2023; Masvotore 2024; Ndabezitha, Mubangizi & John 2024).

Because of its geographic and tectonic conditions, Padang City is highly vulnerable to earthquake-triggered tsunamis. Despite earlier efforts in contingency planning, the city continues to experience fragmentation, evacuation and post-disaster logistics. Key challenges include limited infrastructure readiness, weak communication systems and uncoordinated post-disaster responses. Previous studies have not adequately assessed how infrastructure, communication and logistics failure modes contribute to evacuation and aid distribution risks.

Therefore, this study aims to identify and analyse critical failure points in post-earthquake evacuation and distribution logistics using the failure mode and effects analysis (FMEA) method. The objectives are: (1) to evaluate key risks in evacuation infrastructure, traffic and community behavior; (2) to assess vulnerabilities in post-disaster logistics operations and (3) to propose prioritised mitigation strategies to reduce these risks.

### Literature review

Tsunami evacuation and post-disaster logistics have become central topics in disaster risk reduction, particularly in regions with complex geographic and tectonic conditions, such as Padang City. Previous studies have explored infrastructure vulnerabilities in tsunami-prone areas. Ismail et al. (2020) examined the accessibility of tsunami evacuation routes in Padang. They found that physical infrastructure was neither optimally located nor adequately connected, especially regarding self-supported shelters. Ophiyandri et al. (2022) analysed the readiness of public buildings for temporary vertical evacuation, identifying that available shelters were heavily concentrated in central Padang, with a notable lack of facilities in the northern and southern tsunami red zones. While these studies highlight the physical gaps in evacuation infrastructure, they did not explore failure modes or provide a framework for risk prioritisation.

From a local sociocultural perspective, Alhadi and Sasmita (2016) emphasisd the importance of integrating indigenous knowledge, such as community-based discussions and the leadership role of traditional figures (niniak mamak) in shaping disaster preparedness behaviour among residents of Padang. Nevertheless, their study also revealed that public awareness of evacuation procedures remains limited. Alhadi (2016) further noted that several designated evacuation sites and routes in Padang do not meet the minimum safety and accessibility standards for an effective mass evacuation. Similarly, Syandriaji and Junaidi (2021) pointed out that many residents lack knowledge of evacuation maps and tsunami-safe zones.

On the institutional side, Novert (2016) pointed out that despite the local government having formulated policies to improve community preparedness, its implementation has often been fragmented and under-resourced. Richard (2018) also reported that many designated evacuation buildings in Padang are structurally vulnerable and lack the seismic resilience required for post-earthquake sheltering. These studies indicate that disaster preparedness in Padang is influenced not only by physical infrastructure but also by policy, institutional coordination and public engagement.

Methodologically, the FMEA has been applied in various contexts to evaluate disaster-related risks. Chin et al. (2009) used FMEA uses a group-based, evidence-based reasoning approach to assess potential failures in disaster response systems. Their study showed that FMEA could prioritise multiple risks by considering severity, occurrence and detection. Alruqi et al. (2021) extended the application of FMEA in disaster logistics by integrating structured risk assessment into post-disaster recovery planning. However, these applications are primarily theoretical or conducted in non-coastal contexts, such as industrial or health-related settings, leaving a gap in real-world, localised applications of FMEA in tsunami-prone areas such as Southeast Asia.

In the Indonesian context, existing studies have focused on either evacuation or logistics independently, with limited efforts in integrating both aspects. For instance, Redi et al. (2021) explored hybrid aerial and ground vehicle routing for post-disaster assessment but did not connect it to evacuation dynamics. Masvotore (2024) emphasised the role of faith-based institutions in disaster awareness but did not consider coordination mechanisms or logistical failures. This fragmented approach hinders a comprehensive understanding of disaster response. Thus, this study aims to fill the gap by applying FMEA to tsunami evacuation and post-disaster logistics in Padang City, integrating technical, social and policy-based failure risks within a single analytical framework.

## Research methods and design

This research adhered to recognised ethical standards and was approved by relevant local authorities. Informed consent was obtained from all participants beforehand, guaranteeing their voluntary participation and the confidentiality of the data collected. This study utilised primary data collected through structured questionnaires and interviews. Respondents were selected using purposive sampling, which allows for selecting individuals with specific knowledge or experience in evacuation and logistics processes (Adji et al. 2023). Purposive sampling is a non-probability method most effective when studying a specific cultural domain with experts (Tongco 2007). This sampling method is also called judgemental, selective or subjective sampling (Adji et al. 2021; Crossman 2020).

Respondents were drawn from institutions directly involved in disaster management, logistics and health services. These included the Regional Disaster Management Agency (BPBD), the Department of Transportation and the Department of Social Affairs in Padang. The selection criteria required that participants had been involved in managing the earthquake events in Padang City in 2007, 2009 and 2010. Based on these criteria, 20 qualified respondents were identified, consisting of 10, 5 and 5 personnel from the BPBD, the Department of Transportation and the Department of Social Affairs, respectively.

The interviews were conducted face to face at the respective institutional offices between September and October 2023, with each session lasting approximately 30–60 min. The data were analysed using the FMEA method, a systematic approach to evaluate potential failure modes within a system. FMEA assesses the likelihood and impact of failures, providing a structured process for understanding failure modes and their effects (Alruqi et al. 2021; Ma & Pan 2021). The FMEA evaluates risks based on three factors: Occurrence (O), Severity (S) and Detection (D), each rated on a 1–5 point Likert scale to measure the frequency of each failure mode, the ease of detecting failure mode and its potential severity. A score of 1 represents the lowest risk value, indicating low risk occurrence, severity and detectability. Conversely, a score of 5 indicates high risk occurrence, severity and detectability. A Likert scale measures an individual’s or group’s opinion on a phenomenon, where responses range from low to high intensity (Anjaria 2022; Cheng et al. 2021; Lozano & Taboada 2021; Munawar et al. 2021a). The risk priority number (RPN) is calculated using the formula RPN = O × S × D. Risks with the highest RPN values are prioritised for mitigation (Kim & Zuo 2018). In the FMEA framework, ‘Occurrence’ refers to the likelihood of a failure mode occurrence, ‘Severity’ denotes the impact of the failure and ‘Detection’ reflects the ability to detect the failure (Chin et al. 2009). Kmenta and Ishii (2000) offer an alternative interpretation, defining occurrence as the probability of the failure cause, detection as the inability to detect the failure cause and severity as the failure mode’s impact on the customer.

In this study, potential failure modes were categorised into five key parameters to structure the risk assessment process: (1) infrastructure and evacuation routes, (2) traffic conditions, (3) community and social behavior, (4) information and communication systems, and (5) institutional and policy frameworks. These categories were derived from prior research (Ismail et al. 2020; Noviana et al. 2021) and Preliminary consultations with disaster management officials. This categorisation allows for systematically identifying physical and organisational risks, ensuring that the FMEA process addresses the technical and human factors.

### Ethical considerations

Ethical clearance to conduct this study was obtained from Universitas Andalas (No. 463/UN16.17/PT.01.07/2024).

## Results

### Analysis of failure in the post-earthquake disaster evacuation process

Six risk events were identified in the context of road infrastructure: insufficient capacity of evacuation routes, poor road conditions, inadequate pedestrian facilities, limited access to remote evacuation routes, earthquake-induced damage to evacuation routes and lack of alternative evacuation routes. Among these, the most frequent risk event is the insufficient capacity of evacuation routes (3.13 out of 5). The risk event with the most severe impact is the earthquake-induced damage to evacuation routes (3.88 out of 5). Meanwhile, the poor condition of pedestrian facilities was identified as the most easily detectable risk (3.75 out of 5). Particular attention should be directed towards earthquake-damaged evacuation routes, as they pose the greatest threat to successful evacuation. This priority is given because of their high impact on the community’s evacuation process and low detectability characteristics, complicating public awareness in assessing the usability of evacuation routes.

Under the category of traffic conditions, two risk events were identified: difficulty evacuating because of heavy traffic and the disruption of the evacuation process, hindered by vehicles moving against the designated traffic flow. Both events have an equal impact on evacuation failure, each scoring 3.88. However, vehicles moving against the designated traffic flow are considered the most frequent (4.13) and among the most difficult to detect (2.88).

Within the social and community behaviour parameters, several identified risk events include: individual bringing excessive personal belongings during the evacuation, attempts to pick up other family members, the use of private vehicles, lack of knowledge about designated evacuation routes, unaware of tsunami-safe zones, lack of evacuation facilities for people with special needs and lack of community involvement in disaster mitigation. The most frequent risk was individuals attempting to pick up other family members during evacuation (3.38), followed by a lack of knowledge about evacuation routes (3.25). The lack of knowledge about evacuation routes also became the risk with the most significant impact on evacuation failure (4.25) and the most easily detectable risk (3.86).

In the domain of information and communication parameters, seven risk events were identified: lack of clear signage on evacuation routes, non-compliance by key stakeholders during evacuation, disruption of the telephone network following an earthquake, unprepared communication infrastructure that hinders coordination, failure of the logistical information system, limited access to communication technologies in remote areas and power outages that disrupt technological infrastructure. Among these, the most frequently occurring risk event was the disconnection of the telephone network post-earthquake (3.88 out of 5). The risk event with the most significant impact on evacuation failure was unprepared communication infrastructure (4.13). The most easily detectable risk events were the lack of clear signs on evacuation routes (3.86), non-compliance during evacuation and post-earthquake telephone network disruptions.

Under the institutional and policy parameters, five risk events were identified: lack of a formal tsunami disaster conciliation plan, lack of public dissemination of tsunami contingency plans, lack of explicit post-disaster evacuation coordination, no official policy designating specific road segments as evacuation routes and insufficient designated evacuation routes. The most frequently occurring risk event is the lack of socialisation of tsunami disaster contingency plans (3.63). The risk with the most significant impact on evacuation failure was the absence of a policy designating road sections as evacuation routes (3.63). The most easily detectable risk event was the lack of explicit post-disaster evacuation coordination (3.86). The detailed RPN scores for each risk event can be seen in Table 1.

**TABLE 1:** Risk priority number.

Parameter	Risk event		Occurrence	Impact	Detection	RPN
Road infrastructure	a	Roads used as evacuation routes are inadequate (insufficient capacity)	3.13	3.00	3.50	32.81
	b	The condition of the road used as an evacuation route is not good	2.13	2.88	3.63	22.15
	c	The condition of pedestrian facilities is not good	2.25	3.38	3.75	28.48
	d	Access to remote evacuation routes	2.75	3.13	3.13	26.86
	e	The earthquake disaster damaged evacuation routes	2.38	3.88	2.88	26.46
	f	There are no alternative evacuation routes	2.86	3.63	3.38	34.96
Traffic condition	a	It is difficult to evacuate because there are many vehicles on the road	4.00	3.88	3.00	46.50
	b	The evacuation process was hampered by vehicles going against the flow	4.13	3.88	2.88	45.96
Social and community	a	People bring many goods during the evacuation	3.00	3.38	2.86	28.93
	b	People have to pick up other families during the evacuation	3.38	3.75	3.43	43.39
	c	People use vehicles during evacuation	2.63	3.50	3.00	27.56
	d	People do not know which evacuation routes to use	3.25	4.38	3.86	50.63
	e	People do not know the safe areas of the tsunami disaster	2.63	4.25	3.71	41.44
	f	There are no evacuation facilities for people who need	3.38	3.88	3.43	44.84
	g	The community is not involved in disaster mitigation activities in the evacuation process	2.13	3.75	3.29	26.18
Information and communication	a	There are no clear signs on the evacuation route	2.75	3.75	3.86	39.78
	b	There is no compliance by related parties during the evacuation	3.50	3.13	3.86	42.19
	c	The disconnection of the telephone network after the earthquake made it challenging to coordinate the evacuation process	3.88	3.75	3.86	56.05
	d	Unprepared infrastructure leads to an inability to coordinate the evacuation process	3.38	4.13	3.38	46.99
	e	The failure of the logical information system causes difficulties in prioritising the evacuation process	2.63	3.25	3.00	25.59
	f	Remote areas for refugees do not have adequate access to communication technology	3.00	3.50	3.13	32.81
	g	The loss of electricity caused the technological infrastructure to shut down, hampering communication in the evacuation process	3.50	4.00	3.75	52.50
Policy	a	No tsunami disaster conciliation plan	2.50	3.50	3.71	32.50
	b	No socialisation of the tsunami disaster contingency plans	3.63	3.38	3.71	45.44
	c	There is no explicit coordination regarding post-disaster evacuation	2.75	3.38	3.86	35.80
	d	There is no policy to designate road sections as evacuation routes	3.00	3.63	3.29	35.73
	e	The road designated as an evacuation route is still lacking	2.88	3.38	3.29	31.88

RPN, risk priority number.

**FIGURE 5:**
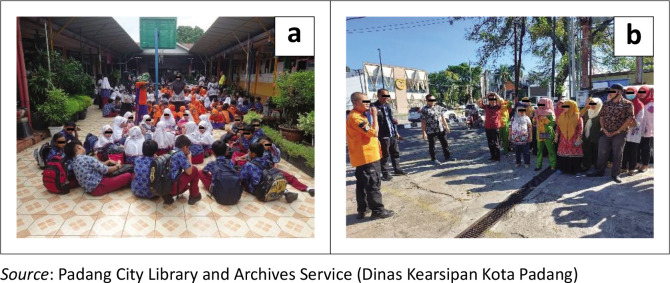
(a) Socialisation of tsunami contingency plans at education centres; (b) Appearance of evacuation simulation preparations in sub-districts.

A conceptual framework is needed to develop an effective tsunami evacuation plan. This framework should prioritise enhancing evacuation route capacity (as illustrated in Figure 5), logistics, supporting infrastructure, controlling the evacuation process during the evacuation and integrating the disaster emergency response command system in post-evacuation resource mobilisation (Lillywhite & Wolbring 2023). Delays and variations in community response during evacuations, identified in various evacuation simulation studies, are often attributed to slow decision-making processes by authorised officials and the lack of reliable, official communication modes for disseminating directives.

### Distribution logistics risk mitigation

Following identifying and analysing all existing risks, the next step involves proposing control or mitigation measures to minimise their potential impact. Risk mitigation efforts are aimed at risks with an RPN exceeding the defined critical threshold. In this study, 13 such risks were identified as requiring intervention. The proposed mitigation strategies were obtained based on experts’ interviews from the BPBD of Padang City, the Transportation Department of Padang City and the Social Department of Padang City. These insights formed the basis for summarising recommended mitigation measures for 11 of the highest-priority risks identified by their RPN values above the threshold.

Table 2 summarises 13 critical risk events identified in the post-disaster logistics distribution process and the corresponding mitigation strategy derived from expert interviews. Key challenges repeatedly emphasised by respondents include post-earthquake communication breakdowns, aid delivery delays and limited vehicle capacity. The BPBD and the Department of Transportation respondents noted the lack of coordination among stakeholders because of damaged communication infrastructure, highlighting the need for alternative communication tools such as radio systems.

**TABLE 2:** Conditions during the distribution logistics process, critical factors and needs.

Kode	Risk event	Needs
G1	Post-earthquake telephone network disruptions hinder logistics team coordination.	Radio communication: Use two-way radios for short-range communication. Backup network providers: Use SIM cards from multiple network providers. Offline maps and plans: Prepare offline maps and routes. Emergency response plan: Have a well-documented emergency response plan. Regular check-ins: Establish regular check-in times to track team status and location.
C3	The duration of logistics delivery is extensive.	Route optimisation: Use route planning software. Inventory management: Ensure goods are available close to demand points. Alternative transportation modes: Consider air transport for urgent deliveries. Predictive analytics: Use analytics to plan for potential delays. Collaboration: Collaborate with other businesses to share transport resources. Technology integration: Use GPS and real-time traffic data.
A2	The vehicles utilised are in subpar condition.	Regular maintenance: Implement a regular maintenance schedule. Vehicle replacement: Replace old vehicles with newer ones. Driver training: Train drivers to detect and report issues. Spare vehicles: Keep spare vehicles available. Quality parts: Use quality parts during maintenance. Driver training: Train drivers to identify and report any issues with their vehicle. Early detection can prevent minor issues from becoming major problems. Spare vehicles: Keep spare vehicles that can be used if the primary cars are not in good condition. Quality parts: Use quality parts during maintenance or repair. They might be more expensive initially, but can save money in the long run by reducing breakdowns.
A1	The capacity of the vehicles used is insufficient for the distribution logistics process.	Vehicle upgrade: Upgrade to larger vehicles. Efficient packing: Implement efficient packing strategies. Multiple trips: Plan for multiple trips if possible. Outsourcing: Consider outsourcing to logistics companies. Collaboration: Collaborate with other businesses. Use of technology: Use logistics management software.
H4	The logistics requirements of the community are not identified.	Community surveys: Conduct regular surveys. Data analysis: Use data analysis tools to predict needs. Community engagement: Engage with community leaders. Feedback mechanism: Provide a feedback mechanism. Regular updates: Provide regular updates on logistics distribution status. Collaboration with local authorities: Collaborate with local authorities.
I1	Adequate storage facilities are lacking at the disaster-impacted location.	Mobile storage units: Use mobile storage units. Shared storage spaces: Collaborate to share storage spaces. Temporary structures: Erect temporary structures like tents. Just-in-time delivery: Implement a just-in-time delivery system. Off-site storage: Store goods at nearby safe locations.
A3	The vehicles cannot traverse the terrain to reach the affected location.	All-terrain vehicles: Use all-terrain vehicles. Air transport: Use helicopters or drones. Local transport: Collaborate with local communities. Improving road conditions: Work to improve road conditions. Alternative routes: Use GPS to find alternative routes. Water transport: Use boats if possible.
I2	The storage post capacity is inadequate.	Expand storage facilities: Expand existing storage facilities. Optimise storage layout: Reorganise the storage layout. Off-site storage: Use rented off-site storage facilities. Just-in-time delivery: Implement a just-in-time delivery system. Inventory management: Implement an effective inventory management system. Use of temporary storage solutions: Shipping containers or portable storage units.
F4	The priorities in post-disaster distribution logistics are ambiguous.	Establish clear guidelines: Develop clear and detailed guidelines. Stakeholder communication: Communicate regularly with stakeholders. Use of technology: Implement a logistics management system. Training: Provide training for all personnel. Regular reviews: Conduct regular reviews and updates. Feedback mechanism: Provide a feedback mechanism for continuous improvement.
B1	The command during distribution logistics is unclear.	Clear communication: Ensure all commands are communicated clearly. Training: Provide training for all personnel. Standard operating procedures (SOPs): Develop and implement SOPs. Feedback mechanism: Provide a feedback mechanism. Use of technology: Implement a logistics management system. Regular reviews: Conduct regular reviews and updates.
A4	Operational support factors, such as fuel supply for distribution logistics vehicles, are inadequately available.	Fuel reserve: Maintain a reserve of fuel. Fuel efficiency: Implement fuel efficiency practices. Alternative fuel vehicles: Consider using alternative fuel vehicles. Fuel contracts: Establish contracts with fuel suppliers. Local suppliers: Identify local fuel suppliers. Fuel consumption monitoring: Implement a fuel consumption monitoring system.

SIM, subscriber identity module; GPS, global positioning system.

The insights from key informant interviews further contextualise these critical risk areas and provide practical field-based evidence supporting the proposed mitigation strategies. Interviews with officials from the BPBD, Department of Transportation and Department of Social Affairs in Padang provided more profound insights into the critical logistical risks identified in the study. Respondents reported that communication failures, particularly the collapse of cellular networks after the earthquake, severely disrupted the coordination of aid distribution teams. This resulted in delayed mobilisation and confusion in the field. In addition, many available vehicles were described as either too small to carry large quantities of relief goods or in poor mechanical condition, further complicating timely delivery. Logistical challenges were also exacerbated by the absence of a clear prioritisation framework, which left field personnel uncertain about which areas should receive aid first. These findings underscore the need for robust communication backup systems, upgraded vehicle fleets and well-defined command and control protocols to improve the effectiveness of post-disaster logistics operations.

These mitigation strategies address technical issues such as vehicle conditions, road access and institutional and communication gaps. The lack of a clear logistical command structure and unclear prioritisation of needs were emphasised across interviews, indicating the need for better inter-agency coordination frameworks during disaster response.

## Discussion

This study identified key risks in evacuation and logistics processes in the aftermath of earthquakes in Padang City. Using FMEA, we found that damaged evacuation routes, limited public knowledge and traffic congestion were the most critical risks, similar to prior research in Japan and the Philippines, where infrastructure and communication failures delayed disaster responses (GeoHaz International 2014; Munawar et al. 2021b). Failure mode and effect analysis proved effective in prioritising risks, aligning with prior applications in disaster management (Alruqi et al. 2021; Chin et al. 2009). Behavioural factors, such as individuals’ intention to gather with family members or confusion regarding the safe routes, also emerged as a disruption for effective evacuation, reflecting the need for studies on social preparedness in tsunami-prone areas (Lillywhite & Wolbring 2023).

Regarding logistics, network failure and insufficient vehicle capacity emerged as the most pressing concerns, confirming earlier reports by Redi et al. (2021) and Ariyachandra and Wedawatta (2023). However, unlike previous studies that examine evacuation or logistics separately, this research integrates both dimensions within a single FMEA framework. This integrative approach distinguishes our study by showing the interdependence between evacuation success and the efficiency of post-disaster aid delivery. Prior studies tended to generalise infrastructure limitations, while this research identifies and prioritises specific failure modes using empirical RPN scoring. Thus, the findings reinforce existing knowledge and offer a structured method for assessing and mitigating local-level risks.

Based on these findings, we recommend several focused actions. Firstly, critical evacuation infrastructure must be reinforced, with redundancies established, particularly in red zone areas that currently lack adequate shelter access. Secondly, public education on evacuation routes and early warning systems must be improved through regular community engagement and simulation exercises. Thirdly, logistics systems should be supported with dedicated communication infrastructure, better vehicle capacity and clearly defined coordination protocols across agencies.

This study is limited by the limited number of respondents and its focus on government stakeholders. To strengthen future analyses, broader engagement with community groups, non-governmental organisations and emergency volunteers is needed. In addition, future research could apply spatial-based FMEA or integrate geographic information system (GIS) modelling to simulate evacuation dynamics and logistics flows. Mixed-method approaches involving community-based participatory risk assessment could also enrich the social dimensions of disaster preparedness. The methodology used here could be replicated in other tsunami-prone cities across Southeast Asia to develop regionally adaptive disaster strategies.

## Conclusion

This study identifies the challenges and risks in managing evacuations and logistics distribution following tsunamis triggered by earthquakes in Padang. Key issues include the lack of integrated evacuation routes, delays in evacuation responses and suboptimal utilisation of transportation resources. Factors affecting logistics distribution include transport reliability, team coordination, security and the readiness of emergency infrastructure. The need for improving evacuation routes in the aftermath of an earthquake emerged as a priority, particularly in response to challenges such as blocked pathways, family reunification efforts during evacuation and limited public knowledge of safe routes. Disruptions to telephone networks and miscommunications about tsunami contingency plans also present significant challenges. Effective disaster management requires emotional support and practical strategies at the household level. Thirty-two potential risks have been identified in the logistics distribution process, covering various technical and institutional aspects. Of these, 13 main risks were prioritised based on their high-RPN values, indicating their frequency, severity and low detectability. These risks include communication failures, unclear directives, delays in aid delivery and limited vehicle capacity. The study improves disaster preparedness by integrating evacuation and logistics in a single risk assessment framework. It offers an approach that can be replicated in other tsunami-prone coastal cities.
